# A Multitarget Gold(I) Complex Induces Cytotoxicity Related to Aneuploidy in HCT‐116 Colorectal Carcinoma Cells

**DOI:** 10.1002/anie.202006212

**Published:** 2020-07-30

**Authors:** Jing‐Jing Zhang, Mohamed A. Abu el Maaty, Henrik Hoffmeister, Claudia Schmidt, Julienne K. Muenzner, Rainer Schobert, Stefan Wölfl, Ingo Ott

**Affiliations:** ^1^ School of Pharmacy China Pharmaceutical University Nanjing 210009 China; ^2^ Institute of Pharmacy and Molecular Biotechnology Ruprecht-Karls-Universität Heidelberg Im Neuenheimer Feld 364 69120 Heidelberg Germany; ^3^ Institute of Medicinal and Pharmaceutical Chemistry Technische Universität Braunschweig Beethovenstr. 55 38106 Braunschweig Germany; ^4^ Department of Organic Chemistry University Bayreuth Universitätsstr. 30 95440 Bayreuth Germany

**Keywords:** aneuploidy, antitumor agents, gold complexes, inorganic medicinal chemistry, metallodrugs

## Abstract

A novel alkynyl phosphane gold(I) complex (trimethylphosphane)(3‐(1,3‐dimethylxanthine‐7‐yl)prop‐1‐yn‐1‐yl)gold(I) **1** displayed mutiple biological activites including selective proliferation inhibitory, anti‐metastatic, and anti‐angiogenic effects. The complex also induced effects related to aneuploidy in HCT‐116 colon carcinoma cells, which might be mainly ascribed to the dysfunction of mitochondrial bioenergetics and downregulation of glycolysis. Induction of aneuploidy beyond a critical level can provide an effective strategy to target cancer, in particular colorectal tumours with a low tolerance of aneuploidy, and could be of relevance for **1** and other metallodrugs.

## Graphical abstract

**Good as gold**: The novel alkynyl phosphane Au^I^ complex **1** displays multiple biological activities including selective proliferation inhibitory, anti‐metastatic, and anti‐angiogenic effects. The complex also induced effects related to aneuploidy in colon carcinoma cells, which may provide an effective strategy to target cancer.

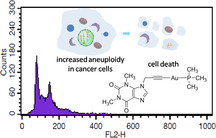

## Introduction

Gold and its complexes have been playing an important role in medicine tracing back thousands of years. Currently, gold(I) compounds are used as therapeutic drugs for the treatment of the symptoms of rheumatoid arthritis; however, ongoing clinical trials focus on the application of gold complexes as anticancer agents and antibiotics. Several cellular targets have been identified, amongst them the selenoenzyme thioredoxin reductase and cysteine‐rich zinc finger motifs, which can interact with the soft Lewis acidic gold centre. The pool of biologically well‐characterized cytotoxic gold complexes has been increasing quickly, confirming the high potential for drug discovery projects. Innovations that might move the field of gold‐based drugs forward towards therapeutic application concern either the investigation of less explored types of biologically stable gold coordination compounds and/or the discovery of novel modes of action.^1^

In this paper we report on the development of an alkynylgold(I)(phosphane) complex that induces cytotoxicity in cancer cells via multiple mechanisms including aneuploidy. To the best of our knowledge, this is the first report on a metal complex acting through aneuploidy induction as part of its mode of cytotoxic action and this finding might have implications for metallodrug development in general. Aneuploidy with abnormal number of chromosomes is one of multiple aspects that make cancer cells different from normal healthy cells. Aneuploidy and hence chromosomal instability (CIN) play a major role in the development of some types of colorectal cancer.^2^

**Figure 1:**
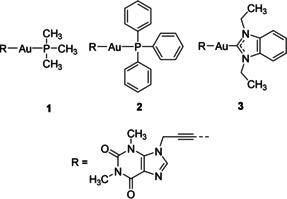
Structures of xanthine‐derived alkynylgold(I) complexes **1**–**3**.

Alkynyl ligands have emerged recently as promising components of gold‐based drugs; however, the resulting alkynylgold(I) species have been much less frequently studied for therapeutic applications compared with other gold complexes.^3^, ^4^ Based on previous works on promising metal‐based drugs with xanthine ligands,^5^, ^6^ the alkynyl group is linked to a caffeine unit in the target compounds described herein (Figure 1).

## Results and Discussion

Complexes **1** and **2** were prepared by reacting 7‐propargyl‐1,3‐dimethylxanthine **4** with the respective chloridogold(I)phosphane complex under basic conditions. Complex **3** was prepared by reacting **4** with chloro‐(1,3‐diethylbenzimidazol‐2‐ylidene)gold(I) **5**. All complexes were characterized by ^1^H NMR spectroscopy and mass spectrometry. Their high purity was ascertained by elemental analyses. Complexes **1** and **2** were readily soluble in dimethylformamide (DMF), in which stock solutions were prepared for application in the biological assays. However, **3** displayed a limited solubility in both organic solvents and water.

**Figure 2:**
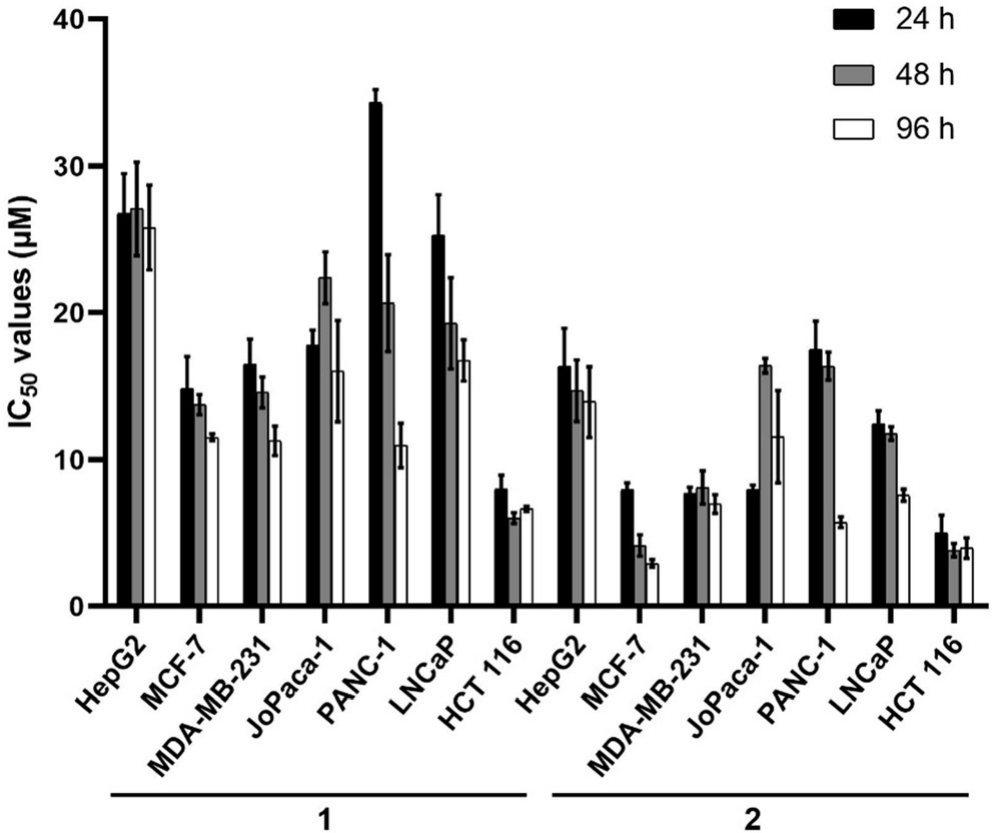
IC_50_ values (μm) of **1** and **2** towards various cancer cell lines (*n*=3). Complex **3** was inactive in all cases (data not shown).

Complexes **1**–**3** were subjected to extended in vitro cytotoxicity studies against various cancer cell lines, including a human hepatocellular carcinoma cell line (HepG2), human breast cancer cell lines (MCF‐7 and MDA‐MB‐231), human pancreatic cell lines (Panc‐1 and JoPaca‐1), a human prostate adenocarcinoma cell line (LNCaP), and the human colon carcinoma cell line HCT‐116 cell line for 24, 48, and 96 h and analyzed with the Sulforhodamine B (SRB) assay. As shown in Figure 2, complexes **1** and **2** displayed time‐dependent cytotoxicity related to cancer types. Highest cytotoxicity was achieved after 96 h of treatment, with IC_50_ values in the range of 6.65–25.8 μm for **1** and 2.93–13.9 μm for **2** (Table S1, SI).

The cell line overall most sensitive to **1** and **2** was the colorectal carcinoma cell line HCT‐116. Complex **3** was inactive against all cell lines studied (IC_50_ values >100 μm), which might be a consequence of the low solubility of this compound. A previously well‐studied biscarbene complex [di(1,3‐diethylbenzylimidazol‐2‐ylidene)]gold(I) iodide **6** was used as a positive reference.^7^, ^8^ It displayed narrow IC_50_ values of 0.14–1.22 μm (Table S1, SI). The 7‐propargyl‐1,3‐dimethylxanthine ligand **4** was used as a negative reference and showed no cytotoxicity even at a concentration of 100 μm, suggesting the crucial role of the gold(I) center for cytotoxicity. The influence of **1** and **2** on a nonmalignant fibroblast cell line for 96 h was also investigated. Complex **1** showed no growth inhibition up to 100 μm, while the IC_50_ value of **2** was 15.3 μm.

In previous reports on different types of gold phosphane complexes we had observed enhanced cytotoxicity and an increased cellular uptake of triphenylphosphane gold complexes compared with alkylphosphane derivatives.^9^, ^10^ However, atomic absorption spectroscopy experiments to quantify the cellular gold content revealed that fibroblast cells could accumulate the trimethylphosphane **1** as effectively as the triphenylphosphane **2** in an 8 h time period, and more effectively than HCT‐116 cells (Figure S1, SI). Hence, the increased toxicity of **2** in comparison with **1** is not the consequence of an increased cellular gold uptake.

Based on the nontoxic behavior of **1** against the nonmalignant fibroblast cells, we selected this compound for further studies on the mechanisms of cytotoxicity in the overall most sensitive cell line (HCT‐116). Solutions of **1** in an acetonitrile/DMF and in an acetonitrile/cell culture medium mixture were evaluated after 6 h by HPLC‐MS (Figure S2, SI) confirming stability of the complex.

**Figure 3:**
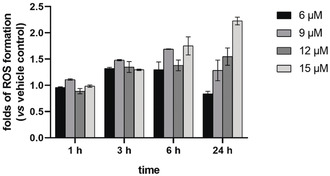
Induction of ROS formation by **1** in HCT‐116 cells after 1, 3, 6, and 24 h of exposure (*n*=3). DMF was used as vehicle control.

**Figure 4:**
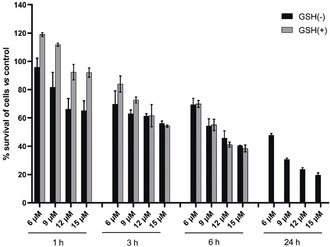
The influence of GSH on the cytotoxicity of **1** towards HCT‐116 cells. Cells were treated with **1** at indicated concentrations for 1, 3, 6, and 24 h, washed with PBS, and further incubated with fresh cell culture medium (GSH(−)) or fresh cell culture medium containing 5 mm GSH (GSH(+)) with a total incubation time of 24 h. (*n*=3).

It has been well established that inhibition of thioredoxin reductase (TrxR) by gold(I) complexes can result in the accumulation of reactive oxygen species (ROS) and trigger cell death via several pathways including the mitochondria‐dependent apoptotic cell death pathway.^4^, ^7^, ^11^, ^12^ The inhibitory effect on mammalian TrxR was first investigated. Complex **1** exhibited strong inhibition with an IC_50_ value of 0.014 μm±0.002, 10 times more effective than **2** (0.110 μm±0.028). The smaller size of the trimethylphosphane ligand in **1** might contribute to an easier substitution reaction with TrxR, resulting in higher activity. As shown in Figure 3, complex **1** also induced a time‐ and concentration‐dependent increase of ROS in HCT‐116 cells after 3 h of incubation. Sustained increase was observed at high concentrations (12 and 15 μm), while there was a decrease at 24 h at the lower concentration (6 and 9 μm). To verify whether the increase of ROS is responsible for the cytotoxicity, after 1, 3, and 6 h of treatment with **1**, the original solution was replaced with cell culture medium with or without 5 mm of the ROS scavenger glutathione (GSH), and the cytotoxicity was investigated after a total incubation time of 24 h (see Figure 4). The cytotoxicity could be significantly reversed when GSH was added at 1 h (before ROS increased), but not after 6 h of incubation (after ROS increased). When GSH was added after 3 h, some reversal of cytotoxicity could be noted for the lower concentrations of **1**. Although the cytotoxicity was not affected when GSH was added after 6 h of treatment with **1**, cytotoxicity generally increased over 24 h, indicating that the increase of ROS is not the only cause of the cytotoxicity.

**Figure 5:**
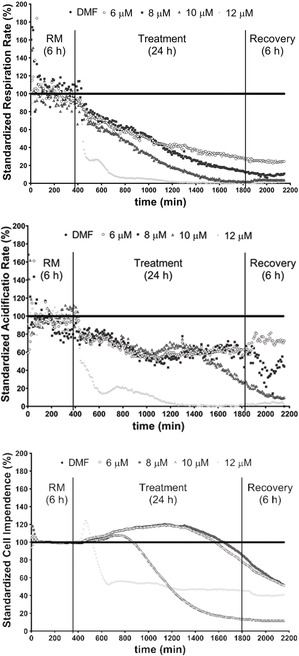
Concentration‐ and time‐dependent effects of **1** on the respiration rate (top), cellular acidification (middle), and cell impedance (bottom) of HCT‐116 cells. (RM: running medium without complexes).

The influence of **1** on mitochondria, which act as both target and source of ROS, was next studied. A decrease of the mitochondrial membrane potential (ΔΨ_m_) was noted after 6 h of treatment by **1** (Figure S3, SI), which occurred after the increase of ROS. This result is in line with previous observations that increased ROS can decrease ΔΨ_m_.^13^ Besides, mitochondrial respiration was immediately inhibited by **1**, as evidenced by the instant decrease of the respiration rate monitored with a Bionas 2500 real‐time biosensor chip system, which can be applied to analyze the metabolism and morphological changes of cells (Figure 5).^14^ This verifies that the dysfunction of mitochondria appeared earlier than the increase of ROS. Moreover, a very sharp decrease of the respiration rate was observed at the concentration of 12 μm, accompanied by a significant decrease of glycolysis and induction of cell death indicated by the predominant decline of acidification and drastic reduction in impedance, respectively. A short and slight increase in cell impedance might be ascribed to the generation of stress fibers.^15^ Similar but more moderate results were found at lower concentrations of **1**. Taken together, these results indicate that **1** may cause cell death via ROS‐independent mitochondrial damage.

Previously investigated gold(I) NHC complexes (NHC=N‐heterocyclic carbene) with phosphane ligands had also shown a strong reduction of cellular respiration, which, however, was accompanied by an initial increase and then decrease of the acidification rate.^7^, ^10^

**Figure 6:**
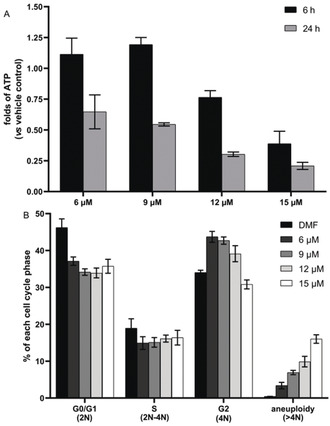
A) the influence of **1** on the ATP levels of HCT‐116 cells; B) DNA content of HCT‐116 cells treated with **1** for 24 h (*n*=3).

Mitochondrial oxidative phosphorylation (OXPHOS) and glycolysis are two major pathways producing adenosine triphosphate (ATP), which is essential for critical cytoplasmic and cellular functions.^16^ Downregulation of these two pathways can reduce the production of ATP. As expected, 24 % and 61 % decrease of ATP levels was observed after 6 h of treatment by **1** at concentrations of 12 μm and 15 μm, respectively, and the decrease reached 70 % and 79 % at 24 h (Figure 6 A). The reduction of ATP production was also evident at lower concentrations (6 and 9 μm) (Figure 6 A). In summary, **1** can significantly reduce the production of ATP, which might be related to its inhibitory effect on the metabolism of cancer cells.

The severe impairment of ATP can affect cellular proliferation and survival in several ways, including dysfunctional cell cycle progression and chromosome separation. Insufficient ATP production is associated with spindle abnormalities and results in aneuploidy.^17^ We therefore analyzed the DNA content of fixed cells with propidium iodide staining and flow cytometry. As depicted in Figure 6 B and Figure S4 (SI), complex **1** induced a concentration‐dependent increase of the cells with DNA contents higher than 4 N, reflecting impaired cell cycle progression and aneuploidy.^18^ The percentage of aneuploid cells reached 16 % after 24 h of treatment by **1** at concentration of 15 μm. It is important to note that treatment severely affected the cell properties in the analysis, which could indicate further severe changes in cell viability. These results are in line with the concentration‐dependent increase of cytotoxicity after the same treatment (Figure 4). Thus, the dysfunction of mitochondrial bioenergetics by **1** may not only impair cell viability but also induce cell death including aneuploidy‐dependent cell death.

**Figure 7:**
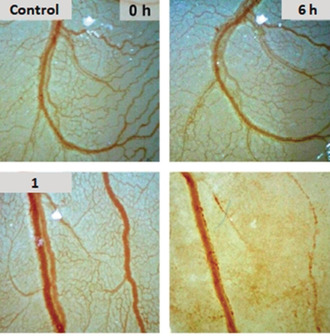
Effects of **1** (10 nmol in 10 μL H_2_O) on vasculature in the chorioallantoic membrane of fertilized chicken eggs after 6 h. Vehicle control (DMF); images are representative of at least three individual experiments.

To further investigate the underlying mechanism, the changes in cellular signalling pathways were investigated using quantitative enzyme‐linked immunosorbent assay (ELISA) microarray analysis.^19^ The analysis revealed a time‐dependent up‐regulation (over 2‐fold) of phospho‐p53 (S392), Akt1 (S473), ERK1 (T202/Y204), and ERK2 (T185/Y187) induced by **1** at concentration of 12 μm (Figure S5, SI).This result is consistent with the above results, as it has been reported that phosphorylation of p53 at S392 can promote the dysfunction of mitochondria and activation of Akt1 can lead to aneuploidy.^20^ Sustained activation of ERK1/2 can also cause cell death, indicating an alternative pathway responsible for the cytotoxicity of **1**. Furthermore, increased phosphorylation was observed for GSK3β (S9) and HIF1α, whereas there were only negligible effects on the phosphorylation of HSP60, and phospho‐p70s6K (T421/S424) was decreased in most cases (Figure S5, SI). In general, the pattern of effects on cellular signaling triggered by **1** showed some similarities to that of a recently studied gold(I) phosphane complex (e.g. up‐regulation of ERK1 and ERK2 phosphorylation).^15^ Besides the cytotoxicity, the anti‐metastatic and anti‐vascular activities of **1** were also investigated, as some xanthine‐derived metal complexes have displayed these activities.^6^ The possible anti‐metastatic effect was studied using highly migratory MDA‐MB‐231 cells by the wound healing assay. The scratch in the cell layer closed much slower after 48 h of treatment with **1** at a subtoxic concentration (2 μm) than in the control experiment (Figure S6, SI). Strong vascular‐disruptive effects of **1** (10 nmol in 10 μL H_2_O) were also observed 6 h after topical application to the chorioallantoic membrane (CAM) of fertilized chicken eggs, indicated by a complete destruction of small blood vessels and distinct hemorrhages of bigger vessels (Figure 7). A similar destruction of the capillary bed in the treated area of the CAM had been observed for ruthenium(II) complexes of the RAPTA type.^21^ An antivascular activity in the CAM assay has also been reported for the ruthenium(II) complex NAMI‐A.^22^

## Conclusion

In this work xanthine‐derived alkynyl phosphane gold(I) complexes were prepared and investigated (Figure 1). Xanthine metal derivatives have recently received increasing attention due to their anticancer, antibacterial, antiangiogenic, and antimetastatic activities.^6^, ^23^ Complex **1** displayed all these activities, including antibacterial effects (data not shown). Such multiple biological activities can strongly decrease the risks of systemic toxicity caused by administration of more than one drug. Moreover, **1** inhibited TrxR, and induced the dysfunction of mitochondrial bioenergetics (increased ROS, and decreased mitochondrial respiration, mitochondrial membrane potential, and ATP level) and aneuploidy in human colon carcinoma (HCT‐116) cells.

Colorectal cancer cells arise from different pathways of carcinogenesis, which leads to a high heterogeneity of these solid tumors. One of the classical carcinogenesis pathways for colon cancer is the chromosomal instability (CIN) pathway that can directly be linked to genomic instability due to the missegregation of chromosomes and aneuploidy. With regard to the different pathways of carcinogenesis, colorectal cancer cells can exhibit a varying degree of tolerance towards aneuploidy.^24^ This tolerance is among other mechanisms also linked to p53. So, in general colorectal cancer cells, such as HT‐29 cells, which are microsatellite stable (MSS), have an activated chromosomal instability (CIN) pathway and, as expected, also harbor p53 mutations.^25^ Therefore, these kinds of cells, should generally have a higher tolerance towards aneuploidy. In contrast, HCT‐116 cells come from the side of the microsatellite instability (MSI) pathway. They do not show a CIN‐activated pathway and have wildtype p53, which fits to a much lower tolerance towards aneuploidy.

**Figure 8:**
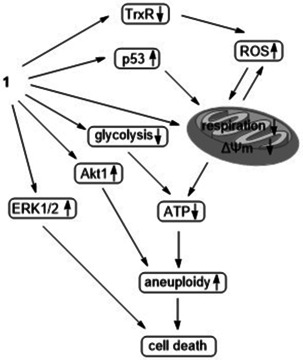
Proposed mechanism of the cytotoxic action of **1**.

To sum up, a newly prepared alkynyl phosphane gold(I) complex **1** displayed multiple functions including selective cytotoxicity, antimetastatic, and antiangiogenic properties. After a detailed study, a mechanism of anticancer action is proposed (Figure 8). Complex **1** inhibits TrxR and mitochondrial respiration and activates ROS and p53 (pS392) in HCT‐116 cells. Together this causes dysfunction of mitochondria. Both the inhibition of mitochondrial activity and the inhibition of glycolysis significantly decrease the ATP level, leading to cell death with aneuploidy together with activation of Akt1. Moreover, a sustained activation of ERK1/2 was also responsible for the cytotoxicity of **1**. Aneuploidy is common in 90 % of solid cancer cells but not in normal cells. A controlled aneuploidy can be lethal to aneuploid cancer cells but safe for diploid normal cells. As a result, this work may inspire the development of novel metal‐based anticancer drugs with improved efficiency. Complex **1** or similar complexes with an identical mode of action represent novel promising therapeutic treatment strategies to target colorectal tumours with a low tolerance of aneuploidy.

## Conflict of interest

The authors declare no conflict of interest.

## Supporting information

As a service to our authors and readers, this journal provides supporting information supplied by the authors. Such materials are peer reviewed and may be re‐organized for online delivery, but are not copy‐edited or typeset. Technical support issues arising from supporting information (other than missing files) should be addressed to the authors.

Supplementary
