# Supplementary material for: A Multitarget Gold(I) Complex Induces Cytotoxicity Related to Aneuploidy in HCT‐116 Colorectal Carcinoma Cells
Source: Angew Chem Int Ed Engl. 2020 Jul 30;59(38):16795–800. doi: 10.1002/anie.202006212 (PMC7540060; doi:10.1002/anie.202006212)
Supplement: Supplementary file 1 — Supplementary [file ANIE-59-16795-s001.pdf]

Angewandte Chemie
Eine Zeitschrift der Gesellschaft Deutscher Chemiker

Supporting Information

A Multitarget Gold(I) Complex Induces Cytotoxicity Related to Aneuploidy in HCT-116 Colorectal Carcinoma Cells

Jing-Jing Zhang, Mohamed A. Abu el Maaty, Henrik Hoffmeister, Claudia Schmidt, Julienne K. Muenzner, Rainer Schobert, Stefan Wölfl,* and Ingo Ott**

anie_202006212_sm_miscellaneous_information.pdf

Electronic Supporting Information (ESI)

Contents:

1. General experimental procedure

A. Materials

B. Instrumentation

C. Synthesis and Characterization

D. Cell Culture

E. Cytotoxicity Assay

F. Cellular Uptake Studies

G. Stability of **1**

H. Mammalian ThioredoxinReductase (TrxR) Inhibition Assay

I. Intracellular ROS Formation Assay

J.The Influence of Glutathione (GSH) on Cytotoxicity

K.Measurement of Mitochondrial Membrane Potential

L. Real-time Monitoring of Cellular Metabolism in Living Cells

M. ATP Quantification Assay

N. DNA Content Analysis

O.Enzyme-linked Immunosorbent Assay (ELISA) Microarray Analysis

P. Wound Healing Assay

Q. ChorioallantoicMembrane (CAM) Assay

2. Figures and tables

3. References

1. General experimental procedure

A. Materials

All chemicals and reagents were purchased from Sigma-Aldrich, unless otherwise noted.

B. Instrumentation

^{1}H NMR spectra were recorded on a Bruker DRX-400 AS NMR spectrometer; Positive-ion ESI (electrospray ionization) mass spectra were recorded on a LTQ-OrbitrapVelos linear iontrap coupled with orbitrap mass analyser (ThermoFisher Scientific); Elemental analyses were conducted in Flash EA112

C. Synthesis and Characterization

(3-(1,3-Dimethylxanthine-7-yl)prop-1-yn-1yl)(trimethylphosphine)gold(I) (1)

A mixture of 7-propargyl-1,3-dimethylxanthine (38.4 mg, 0.188 mmol)(Shenzhen Dieckmann Tech Co., Ltd), potassium *tert*-butoxide (21.0 mg, 0.188 mmol), trimethylphosphinegold(I) chloride (40.0 mg, 0.130 mmol) in MeOH (8 mL) was stirred in dark at 60 °C under N_2 atmosphere for 1 h. After the mixture was dried under reduced pressure, the residue was suspended in CH_2Cl_2 (12 mL) and filtered over Celite. The filtrate was then evaporated under reduced pressure, followed by suspending in CH_2Cl_2. The suspension was washed with H_2O, dried with Na_2SO_4, and evaporated under reduced pressure. The pure product was obtained by addition of *n*-hexane to the CH_2Cl_2 solution of the residue to form a white precipitate. Yield: 21.9 mg (34 %) needle-like non transparent crystals. ^{1}H-NMR (400 MHz, $CDCl_3$): δ (ppm) = 8.10 (s, 1H, C*H*), 5.23 (s, 2H, NCH_2), 3.59 (s, 3H, NCH_3), 3.40 (s, 3H, NCH_3), 1.53 (d, J = 10.2 Hz, 9H, P(CH_3)$_3$). ^{31}P NMR (162 MHz, $CDCl_3$): δ (ppm) =

1.19. ESI-MS (+ve, m/z): 513.1 $[M+Na^+]^+$, 1003.1 $[2M+Na^+]^+$. Anal.Calcd for $C_{13}H_{18}AuN_4O_2P$: C 31.85, H 3.70, N 11.43. Found: C 32.04, H 3.65, N 11.31.

(3-(1,3-Dimethylxanthine-7-yl)prop-1-yn-1yl)(triphenylphosphine)gold(I) (2)

A mixture of 7-propargyl-1,3-dimethylxanthine (60.0 mg, 0.294 mmol), potassium *tert*-butoxide (32.1 mg, 0.286 mmol), triphenylphosphanegold(I) chloride (100.4 mg, 0.203 mmol) in MeOH (16 mL) was stirred in dark at 60 °C under N_2 atmosphere for 1 h. The mixture was then evaporated under reduced pressure. The residue was suspended in around 15 mL of CH_2Cl_2, which was later filtered over Celite. The filtrate was dried in reduced pressure resulting in the crude product. It was purified by addition of *n*-hexane to the CH_2Cl_2 solution of the crude product, which led to the formationof a precipitate. This purification process was repeated until no further pure product was obtained. Yield: 71.2 mg (52 %) needle-like non transparent white crystals. ^{1}H-NMR (400 MHz, $CDCl_3$): δ (ppm) = 8.14 (s, 1H, C*H*), 7.50 (m, 15H, $P(C_6H_5)_3$), 5.27 (s, 2H, NCH_2), 3.59 (s, 3H, NCH_3), 3.40 (s, 3H, NCH_3). ^{31}P NMR (162 MHz, $CDCl_3$): δ (ppm) = 42.34. ESI-MS (+ve, m/z): 699.1 $[M+Na^+]^+$, 721.1 $[M+2Na^+-H^+]^+$, 1375.2 $[2M+Na^+]^+$. Anal.Calcd for $C_{28}H_{24}AuN_4O_2P$: C 49.72, H 3.58, N 8.28. Found: C 49.62, H 3.53, N 8.22.

(1,3-Diethylbenzylimidazol-2-ylidene)gold(I)(3-(1,3-dimethylxanthine-7-yl)prop-1yn-1yl) (3)

The suspension of7-propargyl-1,3-dimethylxanthine (43.9 mg, 0.215 mmol) in MeOH (18 mL) was stirred in dark at 60 °C until it turned into a clear solution. A solution of NaOH (58.5 mg, 0.684 mmol) in MeOH (5.5 mL) was then added, and the final mixture was stirred for 15 min, after which a suspension of chloro-(1,3-diethylbenzimidazol-2-ylidene)gold(I) (86.2 mg, 0.212 mmol) in MeOH was added. Around 5 h later, the mixture was dried under reduced pressure, and the residue was

suspended in 30 mL of CH_2Cl_2, followed by extraction with 15 mL of H_2O twice. After drying with Na_2SO_4, the CH_2Cl_2 layer was evaporated under reduced pressed. The residue was washed with 5 mL of MeOH three times, and was dissolved in a small amount of CH_2Cl_2. The pure product was obtained by addition of *n*-hexane to form a white precipitate. Yield: 57.9 mg, 46.4 %. ^{1}H-NMR (400 MHz, $CDCl_3$): δ (ppm) = 8.23 (s, 1H, C*H*), 7.48 (m, 2H, Ar$H_{5/6}$), 7.43 (m, 2H, Ar$H_{4/7}$), 5.32 (s, 2H, NCH_2CC), 4.55 (q, J = 7.3 Hz, 4H, CH_3CH_2), 3.60 (s, 3H, NCH_3), 3.41 (s, 3H, NCH_3), 1.55 (t, J = 7.3 Hz, 6H, CH_2CH_3). ESI-MS (+ve, m/z): 611.1 $[M+Na^+]^+$, 1199.3 $[2M+Na^+]^+$. Anal.Calcd for $C_{21}H_{23}AuN_6O_2$: C 42.87, H 3.94, N 14.28. Found: C 42.72, H 3.88, N 13.98.

D. Cell Culture

Human hepatoma cell line (HepG2), human breast cancer cell lines (MCF-7, and MDA-MB-231) and human colon adenocarcinoma cell line (HCT-116), human pancreatic cell line (PANC-1), and human prostate adenocarcinoma cell line (LNCaP) were maintained in Dulbecco's Modified Eagle's Medium (DMEM, GIBCO) high glucose supplemented with 10% (V/V) fetal bovine serum (FBS, GIBCO) and 1% (V/V) Penicillin-Streptomycin (GIBCO). Human pancreatic cell line (JoPaca-1) was maintained in RPMI Medium 1640 (GIBCO) with the same supplements as for DMEM. All cells were incubated in a humidified atmosphere at 37 °C under 95% air and 5% CO_2 atmosphere.

E. Cytotoxicity Assay

The cytotoxicities of the complexes toward various cell lines were evaluated by the Sulforhodamine B (SRB) assay according to an established procedure.[1] Stock solutions of **1** were prepared in DMF and diluted with cell culture medium to the indicated concentrations (maximum 0.1 % DMF). Cells (100 µL) were seeded to the

96 well plates with the density of 5,000 cells/ well. After incubating overnight, different concentrations of complexes were added to the plates by serial 1:1 dilution of a 200 µM solution of the complex in cell culture medium. The plates were further incubated for 24 h, 48 h, or 96 h.When the treatment was finished, 50 µL of ice cold 10 % tricholoroacetic acid (TCA) in H_2O was added to each well, and the plates were kept at 0°C for 1 h. After that, the solution in the plates was discarded, and the plates were washed with H_2O twice followed by drying. 100 µL of 1% acetic acid solution containing 0.054 % of Sulforhodamine B Sodium Salt (SRB) were then added. The solution was discarded 30 min later, and the plates were washed twice by adding 200 µL of acetic acid (1%) into each well. The washing acetic acid was discarded quickly every time after washing. The plates were further dried followed by adding 200 µL of 10 mMTris (pH 10.5) to each well to dissolve the SRB dye. After shaking the plates for 15 min, the dye was quantified with a plate reader (TECAN A-5082 AUSTRIA) at 535 nm.The IC_{50} value was determined as the concentration of complexes required to inhibit 50% of cell growth.

F. Cellular Uptake Studies

HCT-116 cells orfibroblast cells were grown in 75 cm^2 cell culture flasks.When the cells had at least 70 % confluence, the medium was replaced with 10 mL fresh medium containing **1** or **2** at the concentration of 5 µM. The flasks were further incubated for up to 8 h. The cells were then washed with 10 mL of PBS, trypsinized, and collected by centrifugation (500 g, 5 min). The cell pellets were collected and further washed with 10 mL of PBS. The final cell pellets were stored at -80 °C for further use. The protein quantification and gold measurements were determined by Bradford method and high-resolution continuum-source atomic absorption spectroscopy (HR-CS AAS) method, respectively, following a reported procedure.[2]

G. Stability of 1

Complex **1** was prepared as stock solution in acetonitrile (ACN). For stability studies in DMF the stock solution was diluted tenfold with DMF and for stability studies in serum free DMEM cell culure medium the stock solution was diluted 1:1 with the medium. For HPLC-MS analysis an Agilent 1620 apparatus equipped with a single quadrupole mass spectrometer (Agilent 6120B) was used. The instrumental setup and chromatographic conditions were as follows: injection volume: 3.0µL; flow rate: 0.8 mL/min; sampler temperature: 37°C, column temperature: 40°C; mobile phase: ACN / ammonium formiate buffer pH 4.0 (10mM) with 0.02% formic acid 1 / 1; stationary phase: ACE UltraCore 2.5 SuperC18 (4.6 x 50 mm, particle size: 2.5 µm); detection wavelength: 280 nm. The identity of **1** was confirmed by MS detection. After 6 h of incubation no significant changes in the peak area of **1** were observed (Fig. S2).

H. Mammalian Thioredoxin Reductase (TrxR) inhibition assay

Mammalian TrxR inhibition assay was performed according to an established microplate reader based assay with minor modification.[3] TrxR from rat liver (Sigma) was diluted with distilled water to achieve a concentration of 3.5 U/mL. To each well of a 96 well plate 25 µL of the enzyme solution, and 25 µL of phosphate-buffered saline (PBS, pH 7.0) solution containing the complex **1** or **2** in different concentrations or DMF as vehicle control, were added. After the 96 well plate had been incubated at 37°C with moderate shaking for 75 min, 225 µL of reaction mixture (1.0 mL consists of: 500 µL of PBS pH 7.0, 80 µL of 100 mM EDTA solution pH 7.5, 20 µL of BSA solution 0.2%, 100 µL of 20 mM β-Nicotinamide adenine dinucleotide 2′-phosphate reduced tetrasodium salt hydrate (NADPH) solution and 300 µL of distilled water) was added. The enzyme catalyzed reaction was initiated by adding 25 µL of 20 mM dithio-bis-2-nitrobenzoic acid (DTNB)

solution in ethanol. The formation of 5-thio-2-nitrobenzoic acid (5-TNB) was monitored by a microplate reader (Perkin Elmer Victor X4) at 405 nm in 35 s intervals for 350 s. A negative control experiment using enzyme free solution was performed to confirm that tested complexes have no interference with the assay components. The residual enzyme activity was determined as percentage of vehicle control ($100*k_2/k_1$, where k_1 and k_2 are the initial rates of enzyme catalyzed reaction in the absence and presence of tested complexes, respectively).

I. Intracellular ROS Formation Assay

Stock solutions of **1** were prepared in DMF and diluted with cell culture medium to the indicated concentrations (maximum 0.1 % DMF). HCT116 cells were seeded in the 12 well plates with 200,000 cells in 1 mL DMEM in each well. After overnight incubation, the medium in each well was replaced with fresh medium containing **1** in different concentrations (6 µM, 9 µM, 12 µM, and 15 µM), and the cells were further incubated for 1 h, 3 h, 6 h and 24 h, respectively. Afterwards, the cells were washed with 1 mL of PBS, trypsinized, and collected by centrifugation (1,600 rcf, 3 min). The cell pellets were collected and incubated in 500 µL of DMEM without phenolred containing 30 µM of dihydroethidium (DHE) in dark for 15 min at room temperature. The cell pellets were obtained by centrifuged at 1,600 rcf for 3 min, and resuspended in 500 µL of DMEM without phenolred. The suspensions were measured with a FACS Calibur flow-cytometer (Becton Dickinson) at 488 nm excitation and 564–606 nm emission wavelength, and analyzed with CellQuest Pro (Becton Dickinson) analysis software.

J. The Influence of Glutathione (GSH) on Cytotoxicity

HCT-116 cells (300 µL)were seeded into the 48 well plates with the density of 15,000 HCT-116 cells/ well. After incubating overnight, the original medium was

replaced with of fresh medium containing different concentrations of **1** (6 µM, 9 µM, 12 µM, and 15 µM) or DMF, and the cells were further incubated for 1 h, 3 h, 6 h or 24 h. The cells, which were treated for 1 h, 3 h or 6 h, were washed with 300 µL of PBS twice. Afterwards, 300 µL of fresh medium with or without glutathione (5 mM) was added to each well. The total incubation time was 24 h. The cytotoxicity was evaluated with SRB assay as introduced above.

K. Measurement of Mitochondrial Membrane Potential

HCT-116 cells were seeded into 12-well plates with a density of 200,000 cells per well in 1.0 mL of DMEM, and incubated overnight. Different concentrations of complex **1** (12 µM and 15 µM) or DMF were then added, and the plates were further incubated for 1 h, 3 h, 6 h and 24 h. Afterwards, cells were washed with PBS, trypsinized, and collected by centrifugation at 1,600 rcf for 3 min. The cell pellets were washed with 1 mL of PBS. The cell pellets were collected by centrifugation at 1,600 rcf for 3 min, and were then resuspended in 500 µL of DMEM (without phenolred) containing 2 µM of (5,5,6,6-tetrachloro-1,1,3,3-tetraethylbenz-imidazolylcarbocyanimeiodide (JC-1) and incubated for 30 min at room temperature in the dark. Afterwards, the cells were collected by centrifugation at 1,600 rcf for another 3 min, and suspended in 500 µL of DMEM without phenol red for FACS analysis at 488 nm excitation, 515–545 nm (FL1 channel) and 564–606 nm (FL2 channel) emission wavelength for JC-1 monomers (green) and JC-1 aggregates (red), respectively.

L. Real-time Monitoring of Cellular Metabolism in Living Cells

Changes in cellular metabolism and impedance of HCT 116 cells treated by gold complexes were analyzed using a Bionas 2500 biosensor chip system (Bionas, Rostock, Germany) as described. [4] In brief, HCT-116 cells were seeded to the chips

at a density of 200,000 cells/chip, in 450µL of full DMEM/ chip. The running medium (RM) used for on-line measurements was prepared from DMEM powder (Pan-Biotech GmbH, Germany) that did not contain glucose, L-glutamine, sodium pyruvate, phenol red or $NaHCO_3$. The medium was then supplemented with HEPES (1 mM), glucose (1 g/L), glutamine (2 mM), FCS (0.1%), and PS (1 %). And for drug activity testing, the following stages were included: (a) 6 h of equilibration with only RM, (b) 24 h of exchange cycles of RM with a freshly dissolved gold complex or DMF, (c) 20 h of recovery with RM only.

M. ATP Quantification Assay

The cellular ATP concentration was quantitatively determined using ATPlite™ 1 step (Perkin Elmer, Germany) according to the manufacturer's instructions. HCT-116 cells were seeded in 96 well black plates with clear bottoms at a density of 3,000 cells/ well, in 100 µL of DMEM/well. After incubating overnight, the medium was replaced with 100 µL of fresh medium containing different concentrations of gold complex or DMF. The plates were further incubated for 6 h or 24 h. Afterwards, 10 mL of substrate buffer solution and 1 vial of substrate solution (lyophilized) were mixed, and 100 µL of the mixture was added to each well of the 96 well plates. The final mixture was mixed well by shaking the plates in a microplate shaker set at 700 rpm for 3 minutes. Luminescence was recorded kinetically for 30 min using a plate reader (TECAN A-5082 AUSTRIA), and results were normalized to the SRB staining of the cells in the same condition.

N. DNA Content Analysis

HCT-116 cells were seeded in 6 well plates with the density of 400,000 cells/ well in 2 mL DMEM. After overnight incubation, the cells were treated with **1** at different concentrations (6 µM, 9 µM, 12 µM, and 15 µM) or DMF for 24 h. Afterwards the

floating cells were collected by centrifugation at 300g for 5 min. The attached cells were washed with PBS, trypsinized, and collected by centrifugation at 200g for 5 min at 4°C. Both parts of the cells were combined and washed with PBS. Cell pellets were obtained after centrifugation at 200g for 5 min at 4°C, which were later suspended in ice-cold 70% ethanol (300 µL) and incubated at -20°C for 24 h. Afterwards, the suspension was centrifuged at 500g for 5 min at 4°C. The pellets were washed and suspended with ice cold PBS containing 1% BSA (m/v), the suspension was centrifuged at 500g for 5 min at 4°C. The cell pellets were then suspended in 100 µL of PBS containing 1% BSA and 50 µg/mL of RNase at 37 °C for 30 min, after which 100 µL of PBS containing 1% BSA and 0.1 mg/mL propidium iodide (PI) was added. The final suspension was mixed well and incubated in dark for 30 min at room temperature, followed by centrifuging at 500g for 5 min at 4°C. The cell pellets were washed with PBS containing 1% BSA, and suspended in 500 µL of PBS. The suspension was analyzed by FACSCalibur flow cytometer (Becton Dickison). Excitation and emission setting were 488 nm and 564-606 nm, respectively.

O. Enzyme-linked Immunosorbent Assay (ELISA) Microarray Analysis

HCT-116 cells (400,000 cells in 2.0 mL DMEM per well) were seeded in 6-well plates and incubated overnight. Then, 2.4 µL of DMF or the 10 mM DMF stock solution of 1 were added to each well. After 24 h of further incubation, the cells were washed with PBS and lysed in 80 µL of lysis buffer (6 M urea, 1 mM EDTA, 5 mM NaF, 0.5% Triton X-100, 10 µg/mL Pepstatin, 0.1mM PMSF, 10 µg/mL Aprotinin, 2.5 mM $Na_4P_2O_7$, and 1 mM Na_3VO_4 in PBS) on ice for 5 min. The supernatant containing protein was collected after centrifugation at 13,200 rpm for 30 min. The proteins were further diluted in dilution buffer (1 mM EDTA, 0.5% (v/v) Triton X-100, 5 mM NaF in PBS, pH 7.2) at a 1:6 dilution, and the corresponding

concentrations were quantified with BCA assay with PierceTM BCA Protein Assay Kit (Thermo Scientific). The expression of phosphorylated proteins were quantified with ELISA microarray based on the ArrayStripTM platform (Alere Technologies GmbH, Jena) following a published protocol. [5]

P. Wound Healing Assay

MDA-MB-231 cells were seeded in 24-well plates. When the cells were almost at 100 % confluence, a narrow artificial wound was created in the cell monolayer by using a 20-200 µL pipette tip. The cell monolayer was washed extensively with DMEM followed by treating with **1**at concentration of 2 µM. The scratch areas were monitored using an EVOS AMG digital microscope after 24 h and 48 h of incubation.[6]

Q. ChorioallantoicMembrane (CAM) Assay

Fertilized, specific pathogen-free (SPF) chicken eggs (VALO BioMedia) were bred in an incubator at 37°C and 60% relative humidity. On day 5 windows (Ø 1.5–2 cm) were cut into the shell at the more rounded pole of the egg. The holes were sealed with tape, and incubation was continued overnight. Then rings of thin silicon foil (Ø 5 mm) were placed on the CAM with its developing blood vessels, and complexes (10 nmol) or the respective amount of DMF as solvent control (all in a volume of 10 µL H_2O) were added. The effects on the developing vasculature were documented after further incubation for 6 h using a light microscope (60× magnification, Traveler).[7]

2. Figures and tables

Table S1. IC$_{50}$ values (μM) of **1**, **2** and [di-(1,3-diethylbenzylimidazol-2-ylidene)]gold(I) iodide **6** towards various cancer cell lines as determined by SRB assays. (n=3).

Cell lines	HepG2			MCF-7			MDA-MB-231		
Time (h)	24	48	96	24	48	96	24	48	96
**1**	$26.7^{\pm 2.7}$	$27.1^{\pm 3.2}$	$25.8^{\pm 2.9}$	$14.8^{\pm 2.2}$	$13.7^{\pm 0.7}$	$11.5^{\pm 0.2}$	$16.5^{\pm 1.7}$	$14.6^{\pm 1.1}$	$11.3^{\pm 1.0}$
**2**	$16.3^{\pm 2.6}$	$14.7^{\pm 2.1}$	$13.9^{\pm 2.4}$	$7.95^{\pm 0.46}$	$4.15^{\pm 0.73}$	$2.93^{\pm 0.25}$	$7.7^{\pm 0.42}$	$8.11^{\pm 1.14}$	$6.98^{\pm 0.64}$
**6**	$10.0^{\pm 0.7}$	$1.35^{\pm 0.65}$	$0.96^{\pm 0.24}$	$6.48^{\pm 0.62}$	$1.47^{\pm 0.19}$	$0.68^{\pm 0.14}$	$5.27^{\pm 0.32}$	$1.33^{\pm 0.13}$	$1.10^{\pm 0.20}$

Cell lines	JoPaca-1			PANC-1			LNCAP			HCT116		
Time (h)	24	48	96	24	48	96	24	48	96	24	48	96
**1**	$17.8^{\pm 1.0}$	$22.4^{\pm 1.8}$	$16.0^{\pm 3.4}$	$34.3^{\pm 0.9}$	$20.6^{\pm 3.3}$	$11.0^{\pm 1.5}$	$25.3^{\pm 2.8}$	$19.3^{\pm 3.1}$	$16.7^{\pm 1.4}$	$8.0^{\pm 0.95}$	$6.02^{\pm 0.37}$	$6.65^{\pm 0.18}$
**2**	$7.98^{\pm 0.30}$	$16.4^{\pm 0.5}$	$11.6^{\pm 3.1}$	$17.5^{\pm 1.9}$	$16.3^{\pm 0.9}$	$5.74^{\pm 0.37}$	$12.5^{\pm 0.8}$	$11.8^{\pm 0.5}$	$7.58^{\pm}$ 0.42	$5.0^{\pm 1.2}$	$3.8^{\pm 0.5}$	$4.0^{\pm 0.7}$
**6**	$14.1^{\pm 3.9}$	$9.53^{\pm 1.9}$	$1.22^{\pm 0.23}$	$2.57^{\pm 0.45}$	$1.19^{\pm 0.29}$	$0.90^{\pm 0.1}$	$1.41^{\pm 0.3}$	$0.93^{\pm 0.28}$	$0.39^{\pm 0.03}$	$2.63^{\pm 0.54}$	$0.13^{\pm 0.00}$	$0.14^{\pm 0.03}$

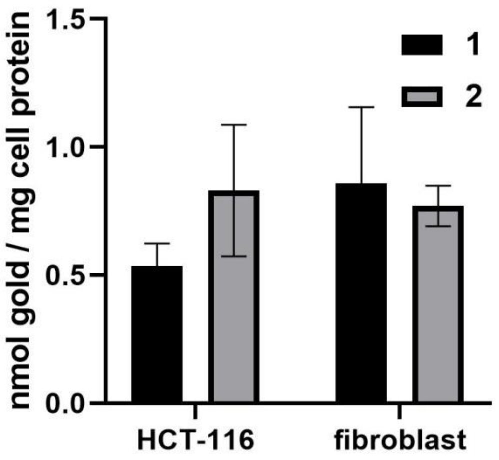

Fig. S1. Gold uptake into HCT-116 and fibroblast cells exposed to **1** or **2** at the concentration of 5 μM for 8 h. (n = 3)

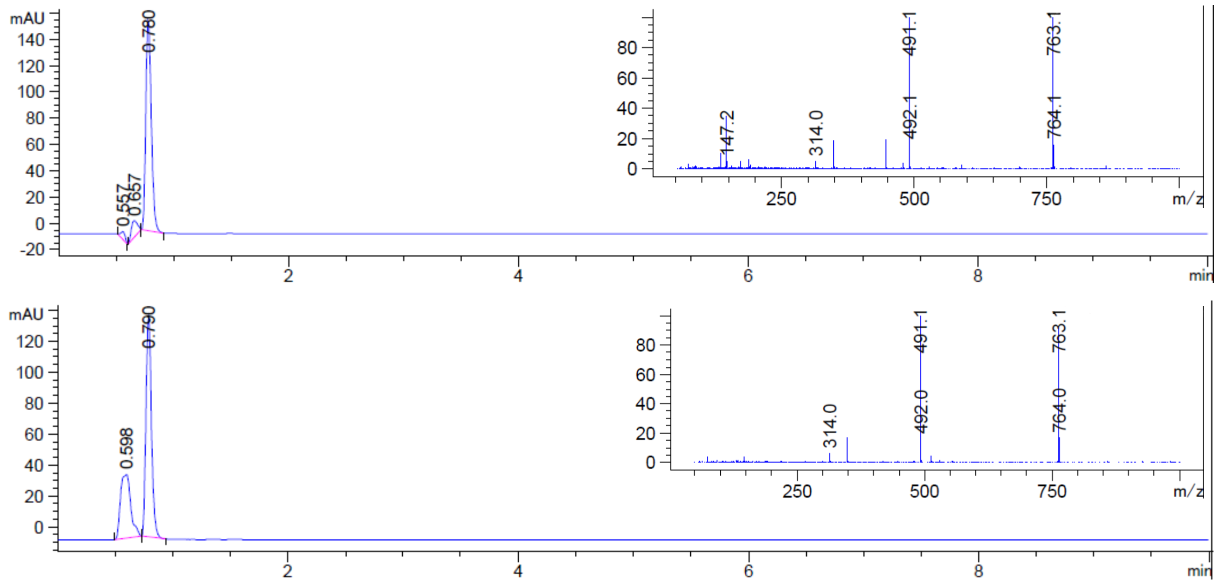

Fig. S2 Chromatograms obtained after 6 h incubation of 400μM **1** at 37°C in ACN / DMF 1 / 9 (top) and ACN / cell culture medium 1 / 1 (bottom). Components of the cell culture medium are eluted at 0.598min (bottom). The inserts show the mass spectra of the peak of **1**.

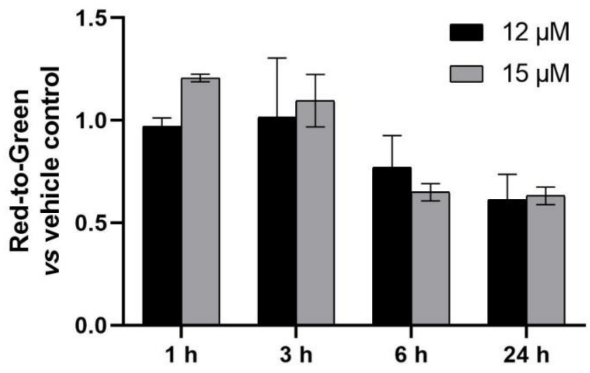

Fig. S3 Complex **1** interferes with the mitochondrial membrane potential in HCT-116 cells. (*n* = 3)

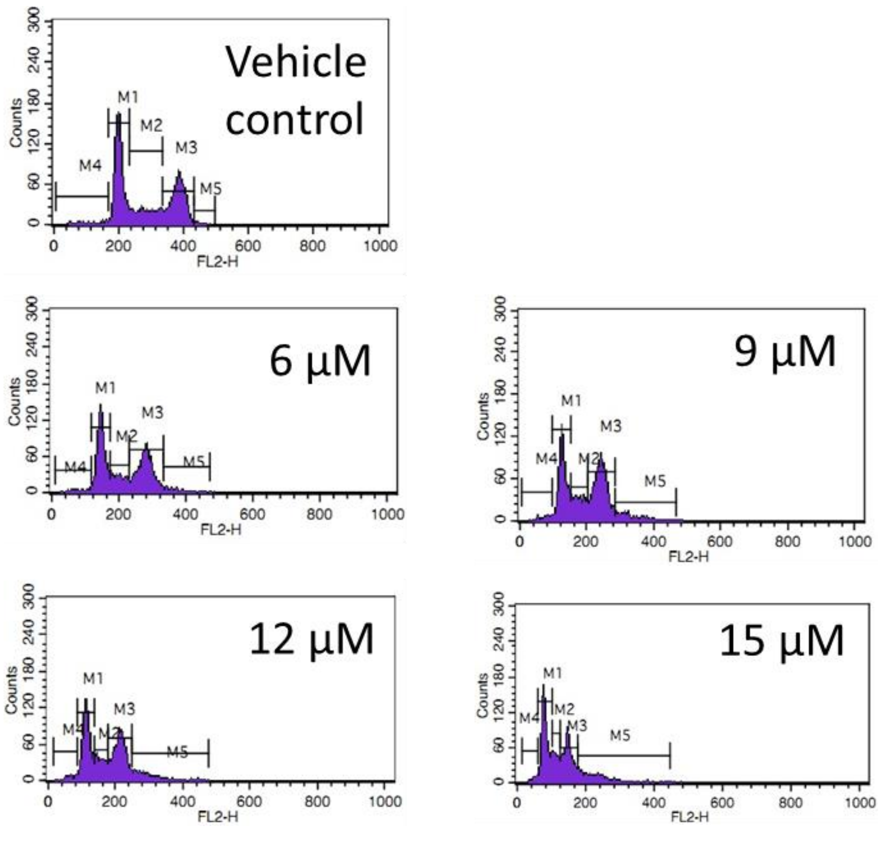

Fig S4 FACS analysis of HCT-116 cells treated with **1** for 24 h (M1: G0/G1 phase; M2: S phase; M3: G2 phase; M4: sub-G1 phase; M5: aneuploidy) (n=3)

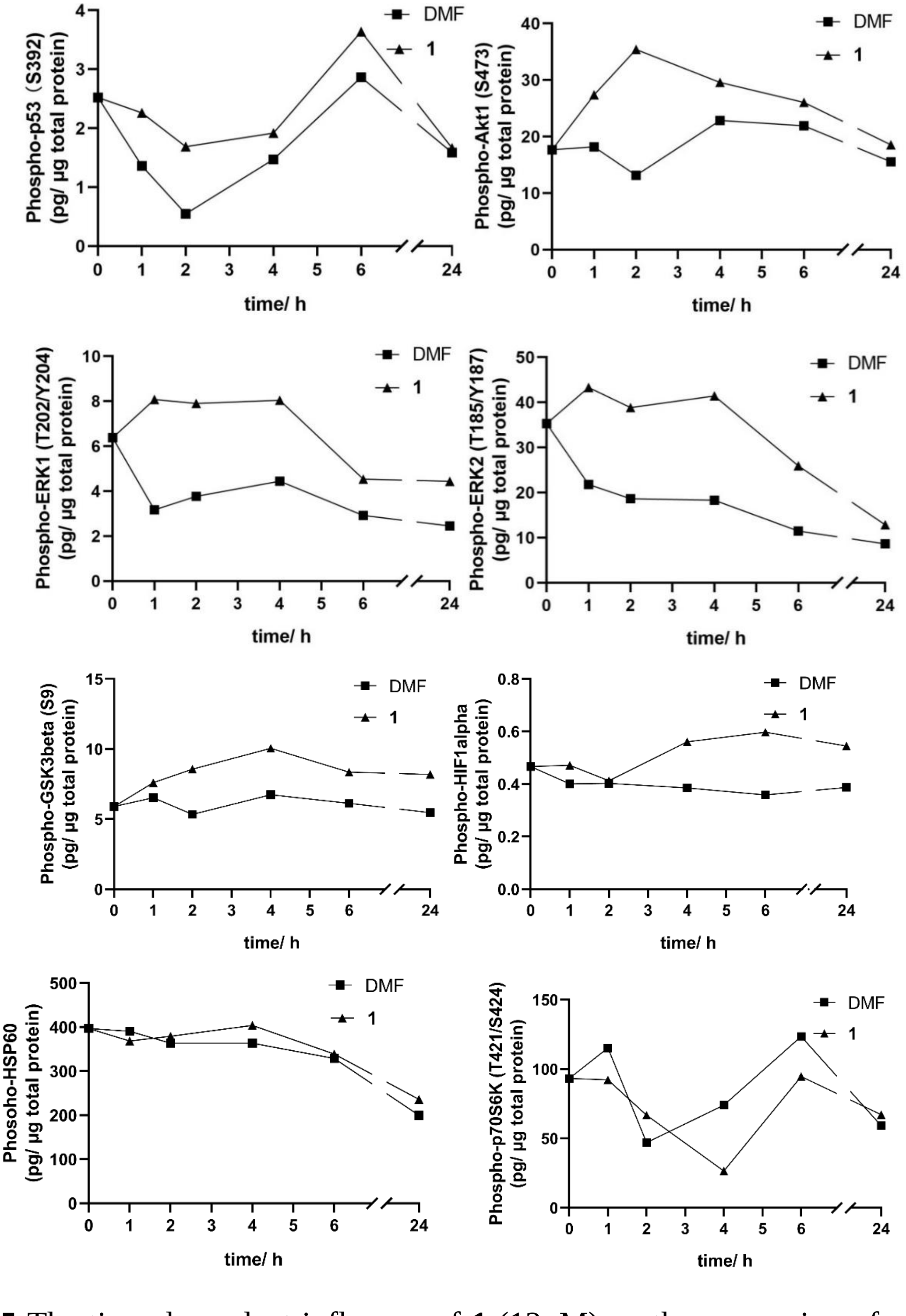

Fig. S5 The time-dependent influence of **1** (12μM) on the expression of phospho-p53 (S392), Akt1 (S473), ERK1 (T202/Y204), ERK2 (T185/Y187), GSK3beta (S9), HIF1alpha, HSP60 and p70s6K (T421/S424) in HCT-116 cells were determined by microarray analysis.

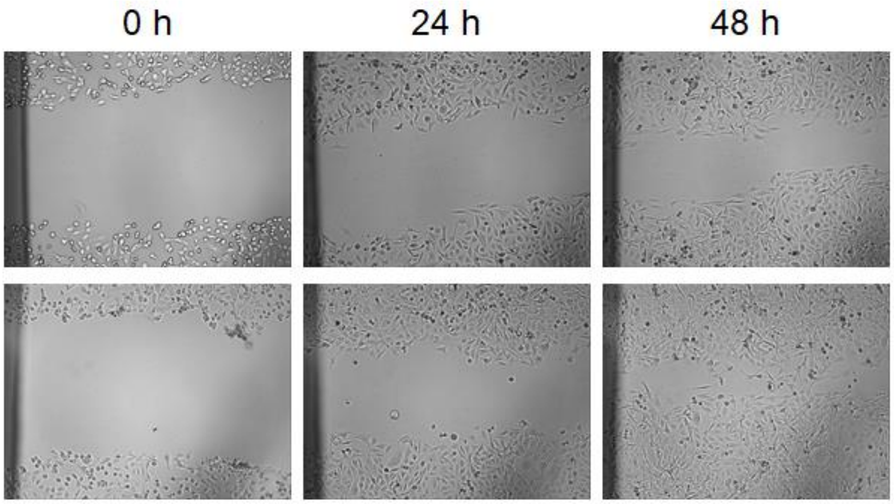

Fig. S6. The comparison of the wound healing capacity of MDA-MB-231 cells treated with **1** (2 µM) or a respective control (DMF).

3. References

[1] J.-J. Zhang, J. K. Muenzner, M. A. Abu el Maaty, B. Karge, R. Schobert, S. Wölfl, I. Ott, *Dalton Trans.***2016**, *45*, 13161–13168.

[2] C. Schmidt, B. Karge, R. Misgeld, A. Prokop, R. Frank, M. Brönstrup, I. Ott,*Chem. Eur. J.***2017**, 23(8), 1869–1880.

[3] R. Rubbiani, S. Can, I. Kitanovic, H. Alborzinia, M. Stefanopoulou, M. Kokoschka, S. Mönchgesang, W.S. Sheldrick, S Wölfl, I. Ott, *J. Med. Chem.***2011**, *54*, 8646–8657.

[4] H. Alborzinia, S. Can, P. Holenya, C. Scholl, E. Lederer, I. Kitanovic and S. Wölfl, *PLoS One***2011**, *6*, e19714.

[5] P. Holenya, I. Kitanovic, F. Heigwer, S. Wölfl, *Proteomics***2011**, *11*, 2129–2133.

[6] C.-C. Liang, A.Y. Park, J.-L.Guan, *Nat. Protoc.***2007**,*2*, 329–333.

[7] a) K. Norrby, *J. Cell. Mol. Med.***2006**, *10*, 588–612; b) B. Nitzsche, C. Gloesenkamp, M. Schrader, M. Ocker, R. Preissner, M. Lein, A. Zakrzewicz, B. Hoffmann, M. Hçpfner, *Br. J. Cancer***2010**, *103*, 18–28; c) J. K. Muenzner, B. Biersack, H. Kalie, I. C. Andronache, L. Kaps, D. Schuppan, F. Sasse, R. Schobert, *Chem. Med. Chem.* **2014**, *9*, 1195–1204.
